# Obsessive-Compulsive Disorder and Myoclonic Movements Following Streptococcal Pharyngitis: A Rare Pediatric Neuropsychiatric Case

**DOI:** 10.1192/j.eurpsy.2025.1707

**Published:** 2025-08-26

**Authors:** J. P. Carrasco Picazo, J. Esteve, C. Conde-Pumpido, B. Herraiz

**Affiliations:** 1 Hospital Provincial Castellón, Castellón; 2 Hospital Clínico Universitario de Valencia, Valencia, Spain

## Abstract

**Introduction:**

Pediatric Autoimmune Neuropsychiatric Disorders Associated with Streptococcal infections (PANDAS) typically describe the sudden onset of neuropsychiatric symptoms, such as Obsessive-Compulsive Disorder (OCD), following streptococcal infections. However, cases that present with comorbid motor abnormalities, such as myoclonic jerks, are rare and pose diagnostic challenges. We report the case of a child who developed severe OCD accompanied by myoclonic movements after a streptococcal pharyngitis infection, representing a rare neuropsychiatric syndrome with an atypical clinical course.

**Objectives:**

To present a rare case of post-streptococcal OCD in a child with comorbid motor myoclonus, highlighting the unusual presentation and the multidisciplinary therapeutic approach.

**Methods:**

An 11-year-old male presented to the emergency department with sudden-onset severe compulsive behaviors, including repetitive prayers and ritualistic actions. These symptoms were accompanied by involuntary, rapid, jerky movements in both upper and lower limbs, consistent with myoclonus. Two weeks prior, the patient had been treated for streptococcal pharyngitis. A comprehensive evaluation was performed, including throat culture, elevated antistreptolysin O (ASO) titers, and electroencephalogram (EEG) to rule out seizures. The Yale-Brown Obsessive-Compulsive Scale (Y-BOCS) was used for assessing OCD severity.

The patient was managed with a multidisciplinary approach involving pediatricians, neurologists, and psychiatrists. A combination of antibiotics, selective serotonin reuptake inhibitors (SSRIs), and clonazepam for myoclonus was prescribed, alongside Cognitive-Behavioral Therapy (CBT).

**Results:**

ASO titers were elevated, indicating recent streptococcal infection, and the EEG showed no epileptiform activity. The initial Y-BOCS score was 32, reflecting severe OCD. After four weeks of antibiotic therapy, CBT, and pharmacological treatment, the Y-BOCS score decreased to 18. Myoclonic movements also reduced by 70% with clonazepam. Symptomatic improvement continued over the next three months, with residual mild OCD symptoms and no recurrence of myoclonus.

**Table:** 

Parameter	Initial Evaluation	After 4 Weeks	After 3 Months
Y-BOCS Score	32	18	10
Myoclonic Movement Frequency	15/hour	5/hour	0/hour
Compulsive Behaviors (0-10)	10	6	2

**Conclusions:**

This case illustrates a rare and complex presentation of PANDAS, where post-streptococcal OCD is accompanied by myoclonic movements. The integration of antibiotics, SSRIs, clonazepam, and CBT significantly improved both OCD and motor symptoms. This highlights the importance of recognizing and treating atypical neuropsychiatric manifestations following streptococcal infections in pediatric populations.

**Disclosure of Interest:**

None Declared

